# Fathers’ experiences of being in change during pregnancy and early parenthood in a context of intimate partner violence

**DOI:** 10.3402/qhw.v11.30935

**Published:** 2016-06-16

**Authors:** Kristin Håland, Ingela Lundgren, Eva Lidén, Tine S. Eri

**Affiliations:** 1 Centre for Women's, Family and Child Health, Faculty of Health Sciences, Buskerud and Vestfold University College, Horten, Norway; 2 Institute of Health and Care Sciences, Sahlgrenska Academy, University of Gothenburg, Gothenburg, Sweden; 3 Faculty of Health Sciences, Oslo and Akershus University College of Applied Sciences, Oslo, Norway

**Keywords:** IPV, fathers, experience, pregnancy, childhood, parenthood

## Abstract

**Objective:**

Intimate partner violence (IPV) is a large public health problem with far-reaching consequences for those involved. The aim of this study was to explore fathers’ experiences of change during pregnancy and early parenthood in the context of IPV.

**Methods:**

The methodological approach in this interview study was hermeneutics, based on a lifeworld perspective. Ten men, who had subjected their partners to violence during the childbearing period, and had become fathers within the previous 6 years, participated.

**Results:**

The analysis revealed four themes: beginning to acknowledge that you are inflicting violence, receiving confirmation that you are more than just a perpetrator of violence, becoming aware of the child, and the desire to receive support in the process of learning how to become a father. Levinas’ concept “the face of the other” is used to interpret the findings.

**Conclusion:**

This study contributes to a more nuanced and expanded picture of IPV. It shows that men who inflict violence want to be and learn how to be fathers. We need more knowledge about how to stop violent acts and support these men in the process of fatherhood.

Intimate partner violence (IPV) represents a major global challenge that affects the health and quality of life of those who are exposed to it. WHO defines IPV as “any behaviour within an intimate relationship that causes physical, psychological or sexual harm to those in the relationship” (Krug, Mercy, Dahlberg, & Zwi, 2002). The prevalence of IPV depends on the definition used. A meta-analysis of 92 studies from 23 countries shows an average for violence against pregnant women of 19.8%. The prevalence of emotional and mental violence is 28.4%, physical violence 13.8%, and sexual violence 8% (James, Brody, & Hamilton, 2013).

Women who are exposed to IPV suffer many health-related problems throughout their life cycle, including death (Ellsberg, Jansen, Heise, Watts, & Garcia-Moreno, 2008; Shay-Zapien & Bullock, 2010). High levels of the symptoms of perinatal depression, anxiety, and posttraumatic stress disorder (PTSD) are significantly associated with having experienced IPV (Howard, Oram, Galley, Trevillion, & Feder, 2013). This can affect a woman's ability to bond and provide caregiving to her child (Shay-Zapien & Bullock, 2010; Taylor, Guterman, Lee, & Rathouz, 2009). Violence in the post-partum period may lead to further maternal psychological distress and depression (Escriba-Aguir, Royo-Marques, Artazcoz, Romito, & Ruiz-Perez, 2013). IPV may also lead to poorer physical health in early pregnancy, including conditions such as urinary incontinence, faecal incontinence and vaginal bleeding (Brown, McDonald, & Krastev, 2008), high blood pressure, severe nausea, vomiting, dehydration, kidney infections, and urinary tract infections (Silverman, Decker, Reed, & Raj, 2006). Women reporting IPV during pregnancy experience more somatic symptoms, report more diseases, and have more hospital visits than other pregnant women (Eberhard-Gran, Schei, & Eskild, 2007; Silverman et al., 2006).

Pregnancy IPV is associated with adverse newborn outcomes, including low birth weight and preterm birth (Bailey, 2010; Han & Stewart, 2014). Violence during pregnancy also poses a higher risk of death (Hall, Chappell, Parnell, Seed, & Bewley, 2014). Research indicates that perinatal distress may lead to structural changes in the brain of the infant, which may cause cognitive impairment, short-temperedness, and restlessness (Burke, Lee, & O'Campo, 2008; Sandman, Davis, Buss, & Glynn, 2012). A review of 23 studies of the impact of perinatal stress found perinatal stress had a small but consistent impact on infant size, early birth, attention-deficit symptoms, and decreased cognitive levels (Talge, Neal, & Glover, 2007). Shay-Zapien and Bullock's synthesis of research examined the impact of abuse on women, foetuses, and developing children and showed that IPV can have both short-term and long-term consequences for the health and quality of life of an unborn child. The child of a woman who has experienced IPV may suffer from its effects without witnessing the abuse, which may have negative impacts on the child's social, emotional, behavioural, and cognitive development (Shay-Zapien & Bullock, 2010).

Research shows that European men want to be involved in pregnancy, childbirth, and childhood. This can positively influence the health outcomes of the father, his partner, and children (Donovan, 1995). Becoming a parent involves major life changes. The transition to fatherhood can be experienced by men as being very stressful, but also highly pleasurable and enriching (Chin, Hall, & Daiches, 2011; Donovan, 1995; Draper, 2003; Fägerskiöld, 2008; Finnbogadóttir, Crang Svalenius, & Persson, 2003; Jordan, 1990; Premberg, Hellström, & Berg, 2008). A metasynthesis by Chin et al. (2011) shows that the transition to fatherhood involves a number of different emotional reactions, including a need to identify with the role of father and a redefining of self and the relationship with the partner. Men report a lack of support mechanisms for the new role in early parenthood and that they need more information on parenting, baby care, and relationships in the antenatal period (Deave & Johnson, 2008).

Men inclined to violence are less able to cope with this transition and face greater challenges during pregnancy and parenthood than other men. The experiences of violent fathers are described as being an inner and outer struggle, feelings ranging from failure to a sense of growth. Shame, guilt, and remorse for the harm that they have caused are also feelings reported by violent fathers (Fox, Sayers, & Bruce, 2002; Perel & Peled, 2008). A study of the experiences of men who have subjected their partners to violence showed that they had, despite the violence, a desire to make their children feel secure and to become good fathers (Håland, Lundgren, Eri, & Lidén, 2014). Research has shown that men with IPV histories play an important role in their children's lives. One study indicates that many abusers who are in treatment are concerned about the effect of their violence on their children and the children of their partners and expressed concern about the long-term effects of the violence (Rothman, Mandel, & Silverman, 2007).

There are, around the world, a wide range of treatment and intervention programs for men who inflict IPV. The main purpose of these treatment programmes is to change violent behaviour into non-violent behaviour. Fathers for Change in the United States has shown, in a pilot sample, an initial feasibility of intervention. The results indicate that a focus on men's role as fathers and their wishes for their children may be a powerful motivation for change (Stover, 2013). Research from Finland showed that talking about fatherhood and relationships with children in treatment groups are important motivators of non-violence (Veteläinen, Grönholm, & Holma, 2013).

Men who attend treatment programmes during the transition to fatherhood are in a dual process of change. There is, however, little knowledge about how men experience this intertwined process. Inflicting violence is a very serious act. Responsibility lies with the perpetrator. Nevertheless, it is important to gain more knowledge about men's experiences if we are to be able to understand and provide appropriate assistance and support to parents who are living with IPV. The aim of this study was to explore the experiences of being in change in men who commit IPV in the context of pregnancy and early parenthood.

## Method

The methodological approach used in this study is life world hermeneutics (Dahlberg, Dahlberg, & Nyström, 2008), based on the work of Husserl (1998/1913), Heidegger (2008), and Gadamer (1995/1960). The common ground of these philosophers is that individuals and their living condition can never be completely understood if they are not looked upon as living wholes. Lifeworld-based research attempts to describe everyday experiences in a systematic and methodological way (Dahlberg et al., 2008). Lifeworld hermeneutics is an attempt to understand the world of human beings just as interpretation and understanding are essential parts of existence. According to Heidegger, the hermeneutical processes of understanding and interpreting reveal the hidden meaning of phenomena (Dahlberg et al., 2008). The phenomenon of this study is then *fathers’ experiences of being in change during pregnancy and early parenthood in the context of IPV*.

### Participants and setting

Ten men were recruited from “Alternative to Violence” (ATV). ATV is a professional research and treatment centre for perpetrators of violence and those who have witnessed or been exposed to violence. ATV is a clinic that specializes in the voluntary treatment of violent behaviour. The clinic provides a low-threshold treatment program to a wide range of men who inflict violence. Voluntary treatment, in this context, means treatment that has not been ordered by a court. The majority of the men self-refer. Some of the men are referred by other help or treatment services, and some are referred by child welfare services.

Some of the men in this study had contacted ATV to seek help with their violent behaviour. Others had been ordered to undertake therapy as a condition for being permitted contact with their children. In this way, all the men were “being in change” from a violent behaviour, which is a concept in the aim of this study.

Participant ages ranged from 25 to 40. They had been in therapy from 3 months to 2 years. The inclusion criteria were, in addition to undergoing therapy at ATV, men who had subjected their partner to violence during the childbearing period, who had become a father within the previous 2 years, and who spoke Norwegian. The study touched on highly sensitive topics. Participant recruitment was, therefore, difficult and time consuming. The researchers only managed to recruit two participants in the first 6 months of the study. The inclusion criteria were therefore broadened to include men who had become a father up to 6 years previously.

Socioeconomic status was not specified in the inclusion criteria. Participants, however, spontaneously provided information on their education and employment during the interviews. Education levels ranged from compulsory to higher education, and employment status ranged from none to fulltime employment. Four men lived with their partners. The men interviewed had between one and four children and were fathers to a total of 25 children. Eighteen of these were biological offspring. None of the participants’ partners were pregnant at the time of the interviews were held.

### Interviewing and transcription

Data was collected through in-depth interviews. The interviews lasted between one and one-half and two hours. The interviews were held at the ATV centre. The participants were familiar with the centre, where the interviewer felt comfortable and safe conducting the interviews. The interview question was: *What do you experience as being important in attempting to change violent behaviour after becoming a father?* The participants were encouraged to share their experiences and talk as openly as possible during the interviews. The interviewer listened to the participants and asked follow-up questions. The interviews were audio-recorded and later transcribed. Spontaneous impressions from the interviews were also noted.

The data analysis was based on lifeworld hermeneutics, which constitutes a spiral movement from the whole to its parts and back to the whole again (Dahlberg et al., 2008). The researchers began data analysis by reading the transcriptions and notes in their entirety, to initiate a dialogue with the text and to reveal the meanings that emerged from the studied phenomenon (Dahlberg et al., 2008). Meaning units consisting of one or a few pieces of the data were also identified and compared. Contradictions and similarities were discussed, and themes were developed from the analysis. The analysis focussed on “otherness” to reveal aspects of the phenomenon that were not a part of our pre-understanding. It gave an understanding that went beyond the context of this study. The main interpretation can open up the readers to a deeper understanding that consists of a dialectic movement from parts of meanings to a new whole, a new understanding of the phenomenon. A main interpretation was created, which concluded the interpretation opportunity for the results to be applied in other contexts (Dahlberg et al., 2008). Finally, theory was used to help us obtain a deeper comprehensive understanding of the data and its meaning. Theory also serves the purpose of controlling pre-understanding. Theory therefore helped us to see something other than that given by the natural attitude and thus created a new whole (Dahlberg et al., 2008). The focus of this study was men's experiences of being in change when becoming a father. Their violent actions were, therefore, not specifically explored but rather seen as part of the phenomenon.

How the world is interpreted and understood is based on the researchers’ pre-understanding. Pre-understanding is an intentional structure of feelings and thoughts, which is activated when we regard something as something and which we unreflectively bring with us when reading a text (Dahlberg et al., 2008). Pre-understanding was, in this study, written down before the interview took place. Pre-understanding was critically reflected upon in dialogue with the text and between researchers throughout the analysis process. Our pre-understanding was that inflicting violence is a serious act. We found it difficult to understand that a human being could inflict violence upon someone with whom they were in an intimate relationship. We also found the infliction of violence upon vulnerable individuals, such as pregnant women and children, to be an even more serious act.

### Ethical considerations

The topic of the study includes issues that can relate to the safety of children. The researcher therefore informed the participants in person and in writing, before the interviews began, that she was legally obliged to notify child welfare services of any suspicions she might have that their children could be exposed to violence. The participants were also informed that everything that was said in the interviews was subject to client confidentiality and that they could withdraw from the study at any time. The researcher was eager to master any negative feelings that might arise during the interviews, to prevent the provocation of the men and the incitement of violent behaviour. She strived to adopt an empathic but objective attitude towards the men. The participants were asked, at the end of the interview, about how they experienced the interview situation. They were also given the opportunity to arrange an appointment with a therapist at ATV. The Regional Ethics Committee of South-Norway (No. S-07107a) and the Norwegian Social Science Data Service (No. 16460) has approved the study.

## Results

Four themes emerged from the analysis. The four themes highlight different aspects of the experiences of violent fathers who are in change during pregnancy and early parenthood. The themes are: *In process of denial to confession that you are a perpetrator of violence, receiving confirmation that you are more than just a perpetrator of violence, the time of transition to fatherhood becomes an eye-opener*, and *the desire to receive support in the process of learning how to become a father*. The quotes are attributed to pseudonyms to ensure the trustworthiness of the study.

### In process of denial to confession that you are a perpetrator of violence

Men who have begun to behave violently towards their partners progress to the acknowledgement that they need help and treatment through the development of a conceptual understanding of violence. The participants’ perceptions of violence differ. Some see violence as being purely physical violence, such as punching and kicking. Others understand that violence also involves anger and threats. A number of the men emphasized that they felt no sense of guilt and that they had a “clear conscience” because they had not inflicted physical violence upon their partner.

Violent behaviour affects the way the men see themselves and how others see them. The men used metaphors such as “big bad guy” and “I was treated like a leper again because I was violent” to describe how they thought others perceived them. They used metaphors such as “having a screw loose” and “I was so crazy” to describe their situation. Some even said that they had asked their therapist whether they considered them mad or insane. Most of the men were, however, eager to stress that they did not see themselves as batterers despite using expressions to describe themselves that relate to violence. They found it difficult to take the first step towards seeking help at ATV. They saw themselves as special cases and not the same as the other men who sought treatment at ATV.

I can't quite see myself as one of those batterers … a typical batterer. There's just been a series of unfortunate circumstances that led to things happening the way they did … I've never been involved in any fights. And I probably don't look like a particularly tough guy, either …. (Tom)

Some of the men felt that their partner's previous experiences of violence meant that they interpreted “everything” as violence. They felt that their partner had provoked them and that she was therefore responsible for some of the violence that had taken place. Other men depict violence as a form of quarrelling. One of the men said that he does not like women who are emotional during their menstruation and pregnancy and that he enjoys provoking them:

But you [women] can be malicious creatures when you're pregnant. But I just have my fun with you. I tease you. There's been more than one angry pregnant woman after me! [Laughs] I'm a bit of a lad in that respect … it runs in the … my dad was like that, too …. (Simon)

A number of the men said that battering a pregnant woman was very unacceptable. They furthermore said that they were afraid for and worried about the child in the womb after they had inflicted violence upon the mother. Men whose acts of violence became publically disclosed and who had received court sentences for their actions had developed an insight into the role they had played in the violence. The consequences of violence include denial of visitation rights and restriction of access rights. Compulsory treatment at ATV may also be required before men are allowed to see their children. Letting their children, family members, and friends down also has an added effect, making the need for change even starker.

Being ordered to undergo treatment at ATV challenges the men and forces them to see themselves from the perspectives of others. Interview subjects who had undergone treatment tended to take greater responsibility for their violent behaviour. However, men in this sample who had only been receiving treatment for a few months were more focused on the shortcomings and responsibilities of their partner.

### Receiving confirmation that you are more than just a perpetrator of violence

The men find that violence is a taboo in society, including in the workplace. They also, themselves, see violence as a taboo. The men, despite giving “justifications” for their violent behaviour towards their partners during pregnancy, feel threatened, embarrassed, and shameful when others learn about what they have done.

It takes courage to contact ATV and the men were afraid of how they will be received. It is important that the therapists treat the men with respect so that they feel that they are seen as individuals with many different sides and not just as perpetrators of violence. The men find that the therapists show no shock or disgust but instead reassure them that they have heard and have a great deal of experience with stories of violence. This creates a sense of hope and of the therapists’ confidence in the men's ability to change.

One of the men describes the consideration he is shown as follows: “I felt as if I'd come home to mum, in a way.” What he meant by this was that he was served coffee, was treated with friendliness, and was listened to. He felt that he was acknowledged as a human being who has not only troubles and disappointments but also positive qualities. This gave him the sense of security he needed to talk about his experiences. “They respect me. I felt it. I felt it the moment I arrived here. They respect me and they see me as an individual. Not just as a moron who beats or who has beaten his wife ….” They hope to be treated with respect when they share their experiences with their families and social networks. They, however, initially find that it is difficult to win back the respect of their work colleagues after they learn that they are receiving treatment at ATV. The role of batterer makes them feel that they are ostracized and they, in many settings, feel that they lose the right to express their opinions. Some of the men choose to not disclose that they receive help at ATV. They feel that they will, in some cases, be suspected of other acts of violence.

The men also attach great importance to their children understanding that Daddy is more than just this angry persona but also has good sides. It is very important to them that they receive positive confirmation and love from their children as their father. One of the men's body language and the way in which he expressed himself changed, in an interview, completely when he began talking about his child. The man moved from crying and bending forward while talking about his violent behaviour to straightening himself up, smiling and talking eagerly and willingly about the positive experiences he had had with his child. The acknowledgement of and love of their children is a significant motivator for changing violent behaviour.

But I hope that my children one day, when they're grown up and have kids of their own and everything, can think: “Dad was stupid and did a lot of very stupid things. But he changed as much as he could. He made the best out of it.” I hope they can say “And for that reason I love him.” (Paul)

### The time of transition to fatherhood becomes an eye-opener

The experience of having the opportunity to become a father begins when a pregnancy is confirmed. Some of the men feared that the mother would decide to have an abortion. Others felt joy and anticipation. Some men, however, felt that their life was falling apart. They felt that they are not ready to become fathers and have a need to maintain their attention and their focus on themselves and their needs. The men report that the experience activates stories of their relationships with their own fathers. Many lack a positive role model in their own fathers, which creates insecurity. They, at the same time, want to give their children a story that is different from theirs. Taking part in the birth is, for some men, a turning point at which they become aware that they are responsible for their own lives.

I wept, actually. I shed a few tears when the child came out and that … It was then that I truly realised that I am a father. You know? … I think everyone should experience it. Because then they really see that they have to get a grip on themselves. That they have to take responsibility …. (Tomas)

It is important for the men to spend time with their children. Many only have limited visitation rights after the relationship with their partner breaks down due to violence. Contact with their children represents a key source of motivation for undergoing treatment to this group of men. The level of access they are granted and the setting in which they meet their children, however, affects their motivation to change. Little access to and little contact with their children diminishes the joy of being a father.

It still feels as if everything's being done on the mother's terms. I'm being observed by lots of people, so it's … it's such an odd setting … so … so … I haven't really had any sense of being a dad yet at all. I still feel as if I'm just some kind of babysitter. It feels a bit odd …. (Erik)

Becoming aware of a child also involves being able to provide your child with a sense of security. The participants seemed to draw a clear line between violence against partners and violence against children. They were concerned about not inflicting physical violence upon their partner during pregnancy because they believed it could harm the unborn child. They do not, however, see that making their partners afraid through threats and anger can inflict stress on the unborn child. Seeing their children's fear face-to-face motivates the men to seek help for their violent behaviour.

Sitting there and watching your own child … creeping around like a beaten dog just because you raise your voice … it's unacceptable … because they're afraid of their dad, of the way he reacts: “What's going to happen next?” (Paul)

### The desire to receive support in the process of learning how to become a worthy father

Many of the men, including those who live with their children's mother, feel that they are not given the opportunity to be fathers. Their experience is that it is the woman who decides how the child is raised. A number of the men are only permitted contact with their children under supervision. The relationship between the father and the supervisor can, however, be strained. Some feel that they are being supervised in a situation in which they feel uncomfortable about their role as fathers. They wished they had more guidance.

There is a supervisor there. The mother is just a few metres away. So it feels very insecure and … there's no-one who gives you any kind of guidance. It's just sort of … well, I sit there by myself and try to make the best out of the situation … So I had hoped I could get to know my daughter better with the help of someone who was interested in guiding me …. (Frank)

All the men talk about pregnancy and birth as being demanding times for men. Pregnancy causes hormonal changes in the mother. The men, at the same time, struggle to find their new identity as fathers. This creates tensions between couples. The men feel that support systems focus on the mother and very little help is made available to the father.

It's very much mother-mother-mother during pregnancy … The mother is always going for check-ups and appointments with the midwife. The only time the father is involved is in ultrasound. Otherwise, we're left out …. (André)

The men also said that they would have liked to have had the opportunity to talk to the midwife alone about becoming a father and about their insecurity in the new role. They consider it important that midwives have sufficient knowledge to be able to ask the father about violence. They also believe that this should be a standard part of prenatal care. “It should be a part of their (health care professionals) training … Force them to ask. People are afraid to ask about that kind of thing!”

The men want to be reassured that they will be given help and that what they say will not be used against them later. They feel that having a third person present in discussions between couples is important. This can give both the man and the woman the opportunity to voice and share their experiences of the conflicts within the relationship. The men would like to have an “instruction manual” for becoming a father and information material on violence during pregnancy.

## Main interpretation

The meaning of “Being in change” in pregnancy and early parenthood, is about being put into a new place in the life. From this place, the men's previous views of themselves as a person, man, and father are challenged. In this study, the concept “Being in change,” also refers to a becoming that begins with an awakening and growing awareness of the men's violent behaviour.

To gain a deeper understanding of fathers’ experiences of being in change during pregnancy and early parenthood in the context of IPV Levinas’ concept “the face of the other” was used for interpretation (Levinas, 1993). Seeing the face of the other means encountering humans in their vulnerability and nakedness, which creates an immediate ethical demand for responsibility for the other. The findings of the present study show that the face of the other can entail both being encountered as “the other” and encountering “the other.” Men want to be seen and encountered by others in a manner that recognizes both vulnerability and violent behaviour. They want to be shown a humanity that accommodates feelings, needs, and longings; dignity and respect despite their violent behaviour; and an acceptance that humans have both good and difficult sides. To be seen by others and accepted as a multifaceted individual is a prerequisite for finding the motivation to change.

However, even if being in change begins with a fundamental awareness and acknowledgement of the men's violent behaviour, they draw a distinction between identity and actions, that a person's identity is much more than the violent actions. The men express that violence may be the result of conflicts in a relationship. They therefore feel that the women also have a responsibility for the violence and that violence may also be the result of a series of negative life events. To see the woman as the other was therefore not part of the findings.

Impending fatherhood is a significant perspective for wanting change. The child, in this context, represents “the other.” The most powerful source of motivation to change is the face of the child, which makes an impact on the men's sense of responsibility through the little child's vulnerability. Memories from the man's own childhood may be brought to life. The men have a need to love and to be loved in return. This need is manifested in their wish to be able to be fathers to their children. Taking on the role of father is not something that is automatic. The men, however, want to learn how to take on this role.

## Discussion

The aim of this study was to explore fathers’ experiences of change during pregnancy and early parenthood in the context of IPV. Men who attend treatment programmes for changing violent behaviour, during the transition to fatherhood, are in a dual process of change. Pregnancy is a time of change for both the expectant mother and father. For women, the change process is more clearly defined by their growing stomach. The psychological change process that should prepare them for parenthood, however, goes on inside both of them and materializes in the creation of “emotional space” for the child they are expecting (Raphael-Leff, 2005). Men inclined to violence are more vulnerable during pregnancy and parenthood and may be less able to cope with this transition than other men (Perel & Peled, 2008).

The results of this study show that the men do not see themselves as a typical batterer. They seem to perceive themselves as victims of circumstances. They feel that violence may occur as the result of conflicts in a relationship or because of a series of negative life events such as a difficult childhood. This is in line with the findings of Edin and Nilsson's (2014) study of male batterer identity construction in Sweden. They argue that men construct ways of defending themselves, such as through the use of excuses and explanations and that they use these to adopt a victim position. According to Edin and Nilsson (2014), a victim has the right to defend himself and save face. The men in our study also sometimes blamed their violent behaviour on provocative behaviour of the women in the relationship. Edin and Nilsson (2014) found the same form of victim blaming in their study of violent men's narratives. Stover (2013) highlights the importance of IPV as an intergenerational problem. The “Fathers for Change” intervention reported in this paper allows a father to begin to develop an understanding of how his childhood experiences affect his current behaviour and choices and provides him with tools to cope and parent his child.

To be seen as being more than a batterer seemed to be very important to the men in this study. Their environment condemned violent behaviour. However, the men distinguish between *being a perpetrator* of violence and the act of *perpetrating* violence. They want to be met and respected by others as a multifaceted individual with both good and bad sides. They want others to also see their positive sides. They, however, find that other's reactions to their violent behaviour ignore these aspects of their personality. Similar ways of perceiving oneself are also reported elsewhere. A study of men in batterer groups in Finland and how they talked about fatherhood found that the men separated their role as father from their history of violent behaviour (Veteläinen et al., 2013). In Edin and Nilsson's (2014) Swedish study, violent men described experiences of a strange self or being out of character when they inflicted violence. They also did not see themselves as being perpetrators, due to positioning themselves as victims. They could present themselves as relatively normal men and the violence as an exception. The men in our study also felt that they were not the typical batterer and saw themselves as special cases and different from the other men seeking treatment at ATV.

Emmanuel Levinas’ concept of “the face of the other” is used in this study as a metaphor for actually seeing or visualizing the child and encountering oneself as a child through the eyes of the child. Seeing oneself through the eyes of a child may be about identifying and recognizing oneself through the child. Insecurity, fear, and violence may be emotions and experiences that are a part of the men's own childhood and life history. Witnessing a child's fear activates feelings from their own childhoods, feelings that they do not want to pass on to their own children. The men realize the importance of not perpetuating the intergenerational nature of IPV (Stith et al., 2000). Looking into the face of the child represents, for the men in this study, a turning point. Being present at the birth and seeing their child for the first time creates an awareness of fatherhood and the presence of the child is an important aspect in their motivation to embark on a process of change. The men's desire that the child's fear should disappear can be understood as being an extrinsic and the joy of being loved and liked by the child can be understood as an intrinsic motivation for change (Stockdale, Sinclair, Kernohan, & Keller, 2011).

The participants in this study had a strong desire to be fathers for their children. Achieving this is demanding because their experience is that it is the mother who decides how the child will be raised. Other studies have shown that fathers who are violent towards their partners may have a strong desire to be good fathers to their children. They are keen to be involved with their children and develop and maintain secure and close relationships with them (Perel & Peled, 2008; Salisbury, Henning, & Holdford, 2009; Stover & Spink, 2012). Some of the men in our study have little contact with their children and contact that is under supervision. This situation can be extremely tense. They feel insecure; they do not know how to handle the situation and need guidance and support. Veteläinen, Grönholm and Holma (2013) emphasizes the importance of talking about fatherhood in treatment groups, as this and their children are powerful motivations for many of these men to relinquish violence. A central premise of the “Fathers for Change” intervention is that focusing on men as fathers and increasing the men's feelings of competence and meaning in the parenting role provides a motivation to change maladaptive patterns (Stover, 2013).

Men who are inclined to violence are as unique and individualistic as other men. The findings of this study, however, reflect the findings of other studies of IPV and fatherhood in the Scandinavian countries. Victim positioning, victim blaming, and the wish to be a father to their children are found in the findings of many studies. Levinas’ concept of “the face of the other” was used in this study to conceptualize the meaning of violent men's experiences.

## Methodological considerations

The purpose of this study was to deepen the understanding of violent men's experiences. It is not possible to generalize the findings of this study to violent men as a population, as the participants were all undergoing treatment at ATV. Findings from a qualitative study must be interpreted in relation to the context, time, and place (Dahlberg, 2006). The fact that the findings are contextual does not mean that they have no meaning in other contexts. They must, however, be understood in relation to any new context. One challenge in this study is that the criteria for participation were broadened to include men who had become a father up to 6 years previously. It could be difficult for the men to remember exactly how it was to become a father, but at the same time they could reflect upon their experiences of change in the time of early parenthood.

The nature of the phenomenon is challenging, on both a personal and a societal level. The researchers met regularly and worked closely together to ensure a critical perspective and to avoid personal emotions and normative attitudes being reflected in the interviews and the analysis (Dahlberg et al., 2008). These meetings were also important to help the researchers deal with the emotional burden evoked during data collection and analysis.

## Conclusion

We have studied the insider perspective of one side of the living in a context of IPV; the male side. The results show that the men's experiences are diverse and multifaceted. The “face of the other” metaphor captures important aspects of men's experiences. These include becoming aware of the child, confronting their own childhood in the child's face, and the need to be seen by others as a whole person with both good and bad sides.

This novel knowledge about the experiences of men who inflict violence during pregnancy and childhood may contribute to a more nuanced and expanded picture of IPV. It shows that men who inflict violence want to be and learn how to be a father. We need more knowledge about how to stop violent acts and how to support these men in their process towards fatherhood.

## Clinical implications

The authors of this paper are midwives and public health nurse. Midwives and public health nurses meet men in the transition to fatherhood and through the parenting period. They are not in a position to give treatment for violent behaviour. Their main scope of practice is to support and strengthen the foundation for a healthy pregnancy, childhood, and family life. The findings of this paper give the health care workers important knowledge about how violent behaviour may affect the transition to fatherhood and may be taken into consideration when planning the care.
